# Scandinavian systems monitoring the oral health in children and adolescents; an evaluation of their quality and utility in the light of modern perspectives of caries management

**DOI:** 10.1186/1472-6831-14-43

**Published:** 2014-04-30

**Authors:** Marit S Skeie, Kristin S Klock

**Affiliations:** 1 Department of Clinical Dentistry - Pediatric Dentistry, The Faculty of Medicine and Dentistry, University of Bergen, Aarstadveien 19, Bergen N-5009, Norway; 2 Department of Clinical Dentistry - Community Dentistry, The Faculty of Medicine and Dentistry, University of Bergen, Aarstadveien 19, Bergen N-5009, Norway

**Keywords:** Oral health reporting, Oral health care, Child, Caries epidemiology and adolescents

## Abstract

**Background:**

Recording reliable oral health data is a challenge. The aims were a) to outline different Scandinavian systems of oral health monitoring, b) to evaluate the quality and utility of the collected data in the light of modern concepts of disease management and to suggest improvements.

**Material and methods:**

The information for in this study was related to (a) children and adolescents, (b) oral health data and (c) routines for monitoring such data. This meant information available in the official web sites of the “KOSTRA-data” (Municipality-State-Report) in Norway, the Swedish National Board of Health and Welfare (“Socialstyrelsen”) and Oral Health Register (the SCOR system, National Board of Health) in Denmark.

**Results:**

A potential for increasing the reliability and validity of the data existed. Routines for monitoring other oral diseases than caries were limited. Compared with the other Scandinavian countries, the data collection system in Denmark appeared more functional and had adopted more modern concepts of disease management than other systems. In the light of modern concepts of caries management, data collected elsewhere had limited utility.

**Conclusions:**

The Scandinavian systems of health reporting had much in common, but some essential differences existed. If the quality of epidemiological data were enhanced, it would be possible to use the data for planning oral health care. Routines and procedures should be improved and updated in accordance with the modern ideas about caries prevention and therapy. For appropriate oral health planning in an organised dental service, reporting of enamel caries is essential.

## Background

Basic oral health surveys should provide a sound basis for estimation of the present status and future needs for oral health care of a population. This view was proclaimed as early as in 1987 by World Health Organization (WHO) [1]. Achieving this depends on continuous critical evaluation of the quality of collected data. It is important that such epidemiological data are comprehensible for health administrators, politicians and the public in general.

**Figure 1:**
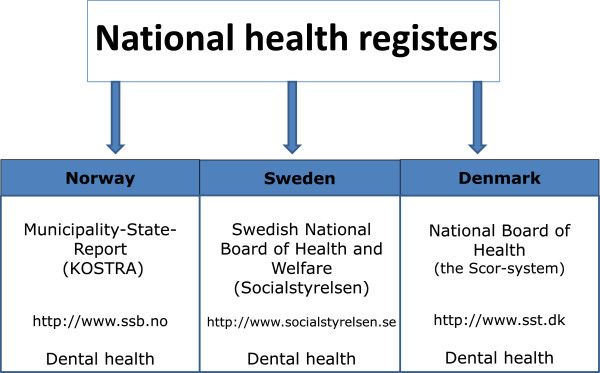
**National Health registers in Scandinavia, which present epidemiological data on dental health.** The registers from each country are here listed, together with the available statistical websites.

The reporting of caries data from child populations has for many years been a research interest and a focus in caries epidemiology. It was the introduction of free dental care for children and adolescents in Scandinavia (Sweden, Denmark and Norway [2]) through Public Dental Services (PDS) which made it possible to collect and report caries data at a national level [3-5]. During the 1970s, the administration of caries data collection in Norway was organized by the National Board of Health and Welfare (“Statens helsetilsyn”). From 2001, caries reporting from PDS was incorporated in “KOSTRA” (KommuneStat-Rapportering/Municipality-State-Report) and made available at Statistics Norway, Figure 1 (http://www.ssb.no). Sweden and Denmark also publish dental health data on websites (http://www.socialstyrelsen.se and http://www.sst.dk). Oral diseases other than caries have been reported much more sporadically or not at all in Scandinavia.

The Scandinavian system of proving free dental care for children and adolescents has been established by law and is provided for all residents in the country. Persons enrolled receive regular dental check-ups. A report, The Nordic Project of Quality Indicators for Oral Health Care [6], showed that in 2009 the proportions of the population younger than 18/19/20-year-olds in Denmark and Norway who used oral health services within a year were 77% and 70%, respectively.

Since the 1960s, intervention strategies have been available to arrest or even reverse enamel caries [7]. This knowledge has influenced the dental treatment strategies [8]. Traditional operative (restorative) treatment alone does not arrest the caries disease, only its symptoms, and it has many disadvantages [9,10]. Non-operative treatment aims to control the progression of caries lesions confined to the enamel. The intervention is two-pronged, consisting of both a local and a general intervention. Remineralisation or fissure sealants are examples of local treatment. General intervention is a strategy to enable the patient to control his own risk factors. The method is to inform about the disease and risk factors and to improve the patient’s oral health knowledge, attitudes and behaviour [10]. Non-operative treatment actually changes the meaning of the traditional “care” concept, as care or treatment is no longer restricted to restorations and extractions only. It might be expected in the future that operative treatment will be chosen as a secondary alternative only when non-operative intervention methods have failed [11].

According to the 2003 World Oral Health Report of caries level of 12-year old children, applying WHO methodology and criteria [12], the Scandinavia countries belong to the so called very low and low-caries countries [13]. Many caries epidemiological studies among young people in Scandinavia have been carried out, including enamel lesions. Longitudinal studies have shown that the rate of caries progression during recent decades has declined, especially in enamel [14]. Consequently, enamel caries increasingly makes up a large proportion of total caries experience. Hugoson et al. [15] have shown that the proportion of enamel caries (initial caries) on approximal surfaces among Swedish 5-yr-olds increased from about one fourth of total caries experience in 1973, to almost a half in 1993. Data from Sweden, published in 2008, showed that initial caries among 15-yr-olds constituted as much as 86% of the total number of carious lesions on approximal surfaces [16].

Another reason why enamel caries is increasing, is that restorative treatment criteria used in Scandinavia over many decades, have changed [17-19]. In three studies (1983, 1995 and 2009), Norwegian dentists were asked about treatment criteria for approximal caries based on drawings which illustrated caries at different radiographic stages and were asked to evaluate whether or not they would restore the lesions. Whereas 66% responded that they would place a restoration in 1983, only 7% would have done so in 2009 [17]. This shift in treatment criteria could explain some of the improvement in dental health seen in Scandinavia, and actually means that the caries decline may have been considerably exaggerated.

The above studies among Scandinavian children and adolescents provide evidence that enamel caries lesions constitute an increasing part of the total caries experience. There exists in Scandinavia therefore a potential for improving oral health by adopting modern treatment strategies. In a well implemented system of dental services, there should be opportunities to put these ideas into practice. It is essential that the quality of caries registrations will be as optimal as possible and that enamel caries will be registered. A prerequisite for planning dental health services is to know the “true” caries prevalence in the area.

The aims of this study were:

• To outline the different Scandinavian systems of oral health reporting;

• To evaluate the quality and utility of the collected data in light of modern concepts of disease management and to suggest improvements.

## Material and methods

An overview of the routines used to monitor oral health data at a national level was made. The information sought was related to (a) children and adolescents, (b) oral health data, and (c) routines for registering such data. Information was extracted from the official web sites in the three countries. Figure 1 illustrates the national health registers which in Norway was the “KOSTRA-data” (Municipality-State-Report), in Sweden the Swedish National Board of Health and Welfare (“Socialstyrelsen”), and in Denmark the Oral Health Register (“the SCOR system” (“Sundhetsstyrelsen Centrale Odontologiske Register”), National Board of Health)).

Initially, a search list of various oral health parameters was constructed for this evaluation to serve as a basis for Scandinavian inter-country comparisons. Parameters investigated were as follows: types of oral disease (*e.g.* caries, erosion, gingivitis and periodontal diseases), other oral health conditions (*e.g.* traumatic dental injuries [TDI]), indices used (*i.e.* crude or more detailed diagnostic systems), indices used (*i.e.* dmfs/DMFS or codes for caries presenting), recording criteria for each disease (*e.g.* enamel caries level or dentine caries level), what is reported (*e.g.* presence or absence of condition (prevalence), severity or distribution of the diseases), and the so called “key age” groups included (*e.g.* 3-, 5-, 6-, 7-,12, 15-, 18-, or 19-yr-olds). The inclusion of certain caries risk determinants (*e.g.* socio-economic and cultural background) was also considered. Within each system alternative oral health registers under development, aimed at improving the existing routines, were investigated.

Additionally, “KOSTRA”, “Socialstyrelsen” and “SCOR” were used as keywords in an electronic search for scientific articles published in Scandinavian journals from 1993 to the end of 2013 (KOSTRA in the Norwegian Dental Journal (N = 9), Socialstyrelsen in the Journal of the Swedish Dental Association (N = 2) and SCOR in the Danish Dental Journal (N = 32)). The same keywords were also used in PubMed for the same time period. “KOSTRA” did not get any result. Seventy-six articles were identified by “Socialstyrelsen” and two articles by “SCOR”, but none involved oral health. For the same period, Mesh term combinations (epidemiology AND oral health AND children) were used in an electronic search for studies published in SweMed database (N = 1). All information relevant for oral health data recording was checked in these articles.

## Results

Most of the epidemiological data on oral health in the three countries concerned dental caries. Table 1 gives an overview of variables and definitions used in monitoring caries, reported by dental health workers in Scandinavia.

**Table 1:** An overview of variables and definitions used in monitoring caries in Norway, Sweden and Denmark

**Variables**	**Countries**		
	**Norway**	**Sweden**	**Denmark**
Age groups being enrolled	5, 12 and 18-yr-olds	3, 6, 12 and 19-yr-olds	5, 7, 12 and 15-yr-olds
Institutions responsible for examinations	PDS*	PDS/PP**	PDS/PP
Sound primary and permanent teeth: (st/ST) = 0, caries free	May have enamel caries, fissure sealants, restorations due to trauma.	As in Norway	Enamel caries reported. In comparing surveys, enamel caries not included
Decayed primary or permanent teeth (dt/DT)	Teeth in need of operative treatment due to caries (d_3_/D_3_ threshold)	As in Norway	Differentiate between a) manifest caries (dentin caries), b) secondary caries, defect or missed filling, and c) chronic caries
Missing primary and permanent teeth (mt/MT)	Teeth extracted due to caries	Not reported	Teeth extracted due to caries (et/MT)
Filled primary and permanent teeth (ft/FT)	Filled teeth, also inlays and prosthetic crowns	Filled primary and permanent teeth (no more details)	As Norway
dmft/DMFT (primary and permanent teeth)	Sum of all teeth in need/have had need for operative treatment, or missed due to caries. Or number of teeth with experience of caries	Missed teeth not included	As Norway
Mean dmft/DMFT or mean dft/DFT	dmft/DMFT 12-yr-olds	dft/DFT 12-yr-olds	deft/DMFT 12-yr-olds
*Frequency distribution*			
Proportion of caries-free (DMFS = 0) individuals	5, 12, 18-yr-olds Caries-free = no active caries (D = 0), or no experience of caries (DMFT = 0)	6, 12, 19-yr-olds	5, 7, 12 and 15-yr-olds
Proportion of individuals, without approximal surface caries DMFSa*** = 0	Not reported	19-yr-olds	Not reported
Proportion of individuals with caries experience on various numbers of teeth	1– 4 teeth, 5–9 teeth, >9 in the groups 5, 12, 18-yr-olds	Similar system in the groups 12, 19-yr-olds	More at surface layer registrations
SiC-index The mean DMFT of third of a population with the highest caries scores	12-yr-olds	12-yr-olds	12-yr-olds

*PDS: Public Dental Service.

**PP: Private Practice.

***a: Approximal surface.

### The data collection systems

Since the beginning of the 1970s, the authorities have collected data from the Public Dental Health Service for children and adolescents in Norway. Systematic reports of dmft/DMFT as a measure of caries experience have been reported from the Directors of the Public Dental Health Service since 1983/84. The national information system and database “KOSTRA” [3] included annual electronic reports of dental health in certain child and adolescent groups. The original dental health information was monitored by dentists working in the Public Dental Service for each county, and the Directors of the Public Dental Service were responsible for completing the electronic forms.

During the period 1985–2005, the National Board of Health and Welfare (“Socialstyrelsen”) of Sweden had published annual overviews of the dental health in child and adolescent populations. Since then, the National Board of Health and Welfare of Sweden had sent survey questionnaires to county councils (“Landstinget”) to get information about dental health status [4,20]. In addition to monitoring dental health data, the county councils gave information about the level of caries risk assessment being carried out. The current dental health reports did not cover children and adolescents from all county councils [21].

In Denmark since the introduction of Danish Child Oral Health Care Act in 1972, the Oral Health Register (“SCOR system”) has been used for child and adolescent populations [5]. Initially, municipalities were responsible for reporting oral health data to a national recording system [22]. These data were reported annually from the municipalities and made available to the Danish statistical office, Statistics Denmark (http://www.dst.dk/en). The register was evaluated in 1997 by a group which also suggested improvements to facilitate machine readable registrations [5]. This led to the introduction of an OCR-blank form (OCR = Optical Character Recognition) [23] which gives extended information about the caries status. The Danish “SCOR system” also includes other oral diseases than caries, such as gingivitis, marginal periodontitis, traumatic dental injuries (TDI), orthodontic diagnoses, hypodontia and oligodontia.

### Inter-country similarities and differences

All three countries used the dmft/DMFT-index [24] when monitoring caries data. The OCR- blank form used in Denmark enabled a wider range of options for caries recording. In this system, there were different codes for initial caries (enamel caries), code 0, manifest caries (dentine caries), code 1, secondary caries, defective or missing filling, code 2, and chronic caries (reporting not obligatory), code 9. In Denmark there is also a different code for missing teeth (m/M) due to trauma or for orthodontic reasons, code 7, while in Sweden missing teeth were not recorded at all. Denmark has a long tradition for monitoring caries and fillings at surface level, but since 1985 caries prevalence has been reported to allow international dental health comparisons. In Sweden, caries at approximal sites was reported in 19-yr-olds while in Norway caries was not, at the surface level. Caries information in the Norwegian system, “KOSTRA”, was not available at the individual level, because the estimates of dmft/DMFT were based on aggregated dental data.

The systems of key groups for oral health monitoring were similar in the three reporting systems. The intention was that all individuals of selected ages should have a dental check. This means that, for example, all 5-yr-old children born in 2005 should be examined during the year 2010.

The most pronounced difference in monitoring caries between the countries was that in Denmark it was obligatory to record initial caries (enamel caries), while in Norway and Sweden only caries at dentine level was included, in line with the criteria recommended by the World Health Organization [12]. Only the Danish “SCOR system” stressed the importance of calibration of examiners in the guidelines. Practice calibration sessions were recommended to be constantly integrated in the quality development of the services. In Denmark, not all dental clinics taking care of children and adolescents were public, but dental health recording was obligatory, even in those municipalities where care was provided by private practitioners.

All Scandinavian countries reported on their oral health workforces, the numbers of licensed and active oral hygienists, dentists and specialists. The total cost of oral health per capita in specific age cohorts was also reported in the PDS. Furthermore, in Norway and Denmark, the proportion of the population under 18/19/20 years that had used dental health services each year was reported [25]. The “key age” groups being examined varied between the countries. Around half of the municipalities in Denmark had chosen to monitor oral health for more than the four obligatory age-groups. This was done to assist service planning. The “SCOR system” also provided a better potential for planning dental care than the other Scandinavian systems.

### Projects under development

In Norway, there is ongoing collaboration between “KOSTRA” and the Norwegian Institute of Public Health and the Ministry of Health and Care Services in monitoring the dental health [26]. Furthermore, since 2005, the Norwegian Directorate of Health (Helsedirektoratet) had promoted the use of quality indicators in the dental health services. This work had resulted in nine indicators, some of which were included in the current Nordic project to develop quality indicators [10]. One proposed indicator, not yet implemented, would register the proportion of 2-yr-olds referred from child health clinics to the PDS [27].

In Sweden, an ambitious register, the Swedish Quality Register for Caries and Periodontitis (SKaPa), is under development and will use information memory storage for transferring patient information to the national registry [28]. Then the traditional registries could be supplemented with additional information about oral health, *e.g.* enamel caries and self-perceived oral health (“Patient Reported Outcome Measures”).

The working group of the project “Quality indicators in oral health care: A Nordic project” [25] had already analysed some of the newly developed quality indicators of oral health care. This work was seen as important because it had the potential to transform the data from describing symptoms of the diseases to including aetiological information and thus leading to prevention of the diseases.

## Discussion

There are many similarities in the three Scandinavian reporting systems for oral health among children and adolescents. The diagnostic criteria for reporting, if not identical, all had their origin in the dmft/DMFT index [24]. The data were from different parts of the countries. However, the collected data in Norway and Denmark were estimated to be more representative than in Sweden, as Swedish reports did not cover the whole nation. Only the “SCOR system” in Denmark reported on enamel caries lesions and this system also included other oral diseases than caries data to some extent.

### Quality of the data

The omission of enamel caries in Norway and Sweden negatively impacted the validity of the collected caries data, a phenomenon addressed by Swedish authors [29,30]. Other aspects were also connected with reduced validity. Recall intervals are normally based on caries risk estimated at individual level with the consequence that time intervals between examinations will differ. For instance, Norwegian data from 2003 have shown considerable inter-county variation in the proportions of 5-yr-olds being examined and treated; the mean was 78% (range: 54–96%) [31]. More recent data, from 2012, for the same age group reflected an even lower proportion with a mean of 73% (range: 54-95%) [32]. A similar proportion (77%) was found among 5-year-olds in the Danish data [33]. It might therefore be questionned whether the data being registered were representative for the whole age groups selected for registrations. Swedish researchers have also discussed validity problems due to missing caries data in the recordings for children from 6 to 9 years of age [34].

As long as the examiners are not calibrated and do not regularly participate in collective training sessions, reliability cannot be optimal. Ongoing discussions in the dental services reveal a growing awareness of this problem, which in many Danish municipalities have resulted in calibration trials. Under- and over-restoration of teeth [35] may partly be explained by the opposing caries treatment concepts among the dental practitioners. Dentists, whose main aim is to treat the caries disease, may be more reticent about placing a restoration until the disease is under control. It has been documented that younger, more often than older dentists, would postpone restorative treatment when confronted with same patient cases [36]. Modifying factors in dental health services were that the caries diagnostic systems and routines for data registrations have been used for many years and were well established.

The limitations of the dmft/DMFT index [24] for epidemiological use have been discussed [37,38]. It is claimed that it mixes disease and treatment [39] and makes it difficult to differentiate between previous or existing caries. The index is irreversible and cannot inform whether restorations (filled teeth (f/F)), are due to caries or other reasons, *e.g.* hypoplasia. The “filled teeth (f/F)” criterion is also inaccurate as the criteria behind the decision of a practitioner to fill a tooth, are undefined. Another problem is that the dmft/DMFT index does not indicate whether the caries lesion reported is in an active or inactive state (arrested caries). It is additionally impossible to consider the number of teeth that are at risk of caries and it cannot monitor caries progression. As the Swedish system does not report “missing teeth (mm/M)”, Scandinavian inter-country comparisons using the dmft/DMFT index are problematic.

### Utility

Compared with the other Scandinavian countries, the data collection system in Denmark appears more functional and has adopted more modern concepts of disease management. Enamel caries is included in the “SCOR system”, and it also allows limited registration of ethnic background (Danish/not Danish). The potential to plan dental care is better than in the other countries. The current systems in Norway and Sweden, without enamel caries recordings, are more adjusted to the times when caries management meant traditional operative treatment. The “caries free individual” outcome term in the “KOSTRA” data statistics in Norway, when enamel caries is excluded, only underlines that the modern caries strategies have not been adopted. It is argued that such terminology is misleading and should be avoided, it only hampers links between clinical and research workers [40].

Socio-economic gradients in oral health prevail among children, including children in countries with publicly financed and organised dental health care [41]. All Scandinavian countries are able to report on geographic gradients in the distribution of caries in the population [42,43], but not on socio-economic gradients.

### Potential for increasing utility

Modern data processing capacity makes it possible to analyse huge amounts of epidemiological data, but authors have argued that these amounts of available data do not appear to be widely used when planning dental care [44]. Nevertheless, some researchers have discovered novel approaches and used them in oral health planning. Swedish researchers in the region of Halland (western coast of Sweden) have developed a system of geo-mapping and found parish of residence to be a caries predictor for preschool children [43]. The purpose of such geo-mapping is to distribute resources to regions most in need of prevention [45]. In Scotland, the “National Dental Inspection Program” has linked caries prevalence data from different geographical regions to existing social background data. Valuable caries risk information about the populations studied has been gained in this way [46].

**Figure 2:**
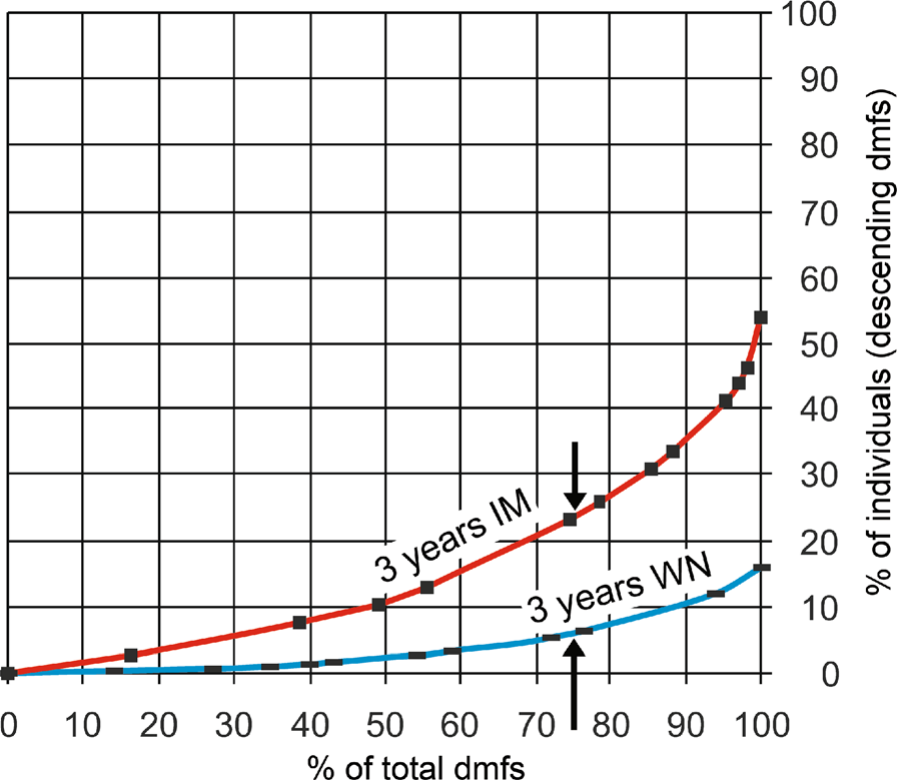
**Lorenz curves for caries distribution in two 3-year-old groups, illustrating deviation in caries skewness.** Footnote: *Each point on these Lorenz curves denotes the proportion of the population (y-axis) responsible for the proportion of the total burden of caries lesions (x-axis). The arrows indicate that 6% of a western native (WN) of 3 year-olds and 23% of an immigrant group (IM) of non-western origin, 3 year-olds, carry about 75% of the total caries burden. This graph is adjusted from an article based on this survey *[48] .

In Denmark, Poulsen et al. have used national DMFS data of 15-yr-olds to describe the distribution of the “total burden” of caries [47] using Lorenz curves. By describing caries skewness in populations, these distribution curves are useful in determining the appropriateness of implementing high-risk preventive strategies. The Lorenz curves in Figure 2 shows the cumulative distribution of caries lesions in 3-yr-olds with western and non-western background, living in Oslo in 2002. Each point of the curves illustrates the proportion of the sample (y-axis) responsible for the total caries experience (x-axis). The arrows indicate that 6% of the western group and 23% of the immigrant group of non-western origin, carry about 75% of the total caries burden. This implies that the distribution of caries (enamel lesions included) is more skewed in the group of 3-year-olds of western-ethnic origin than in the similar age group of children with non-western background [48]. This difference in distribution may indicate that the same preventive strategies do not work equally well in both populations, and may have to be tailored [49]. The Lorenz curves are therefore a useful supplement to the SIC-index (mean of DMFT for one third of the population with the highest caries score) which is already in use [50]. If the SIC-index is used alone [51], the Lorenz curves contribute additional relevant information.

Sheiham and Sabbah in 2010 [44] suggested that trend lines for caries in cohorts are useful tools for predicting future caries development. High value of caries experience in a cohort at baseline is one predictor of future high caries increment [16]. Whether the individuals belong to a population with high or low caries prevalence might also be judged by which tooth groups, teeth or tooth surfaces are most caries prone [52]. In a low caries population, a lower proportion of buccal, lingual and approximal carious lesions in relation to the DMFT index can be expected than in a high caries risk subpopulation. This information is valuable for targeting preventive strategies. It assists the choice between targeting approximal sites for fluoride varnishes or occlusal surfaces and fissure sealants. The ratio of enamel caries to dentine caries is another tool which may indicate the risk of caries progression in the population; a higher value could be expected in a population with low caries prevalence compared with high [53]. Lastly and importantly, signs of enamel caries represent an important caries predictor [54], and should be used as a tool in planning dental care. If an especially high prevalence of enamel caries is found in a group of adolescents, it means that if no non-operative preventive treatment is done, the caries will probably progress into dentine [16]. All these predictions enable us to distribute dental personnel to groups in need for prevention and non-operative treatment. It is important to note that the predictions allow us to plan number and type of dental personnel required, not which child needs therapy [44]. It is also worth mentioning that as long as enamel caries prevalence is unreported, dental health policymakers and authorities will wrongly be informed about the magnitude of caries disease and deprived much of the incitement for prevention. This is worrying, as they are responsible for planning and financing the health care for children and adolescents.

### Suggestions for improvement

As enamel caries among adolescents progresses slowly [55], it should not be necessary to register caries in these groups every year. In contrast with the permanent dentition, in the early primary dentition, caries progression is more rapid [56] and the existence of deciduous tooth caries lesions is a recognised predictor of future caries development [16]. Epidemiological caries mapping of younger children should therefore be prioritized. Caries reports of randomly selected samples of 5-year-olds should be undertaken annually. Only trained and calibrated examiners from national meetings should undertake the examinations. Such expert groups should be trained collectively and calibrated diagnostically in other oral diseases. The clinics from the different counties and the patients should be randomly selected. Such an alternative data reporting system would improve the validity and reliability of the data. For experienced examiners, to allow assessment of the levels of enamel caries, does not adversersely affect the reliability or benchmark validity to a significant degree [57].

Such complex oral health examinations will mean additional cost-benefit analyses, and the balance of cost against the benefits has to be evaluated. If high quality data enable us to plan more precisely future oral care and target the use of resources for prevention, the results may be fewer resources used in the treatment of dental diseases and their sequelae. On the other hand, due to cost reductions in the automation of transfer of data, discussed by SKaPa [28], and a reduction of annual examinations for some key groups, should contribute to expenditure reductions.

Planning of care in some high caries risk groups should include children younger than five years of age, possibly recruited through cooperation projects with child health care clinics. The universal “lift the lip” program has been shown to be capable of detecting signs of caries in very young children [58]. In spite of the fact that the quality of the examinations cannot reach that achievable with older children, this screening could be used as a supplementary tool in some sub-populations of 1- and 2-year-olds. Parents should be invited to complete a questionnaire concerning their attitudes to dental health and their care routines for their children’s teeth. For children with an immigrant background, the country of origin is important information [59].

The present evaluation of the current systems of reporting oral health data shows that promising projects are under development, such as the Nordic project of developing quality indicators of oral health for epidemiological use [25]. The working group responsible for project implementation, has stated that the future focus should be to develop indicators more precisely connected to quality. In spite of this, the proceedings, formulated in 2012, state that caries should be recorded at the D_3_ threshold. Hopefully, this will be altered because such data only give an estimate of the number of teeth that have failed to be treated in the optimal option (non-operative treatment). Given that enamel caries is reversible, the opportunity to monitor regression of initial caries lesions in longitudinal studies [60] dealing with preventive strategies, is missed.

## Conclusions

The different Scandinavian systems of oral health monitoring have much in common, but it appears that the “SCOR system” in Denmark has adopted more modern concepts of disease management than the other systems. There exists a potential for enhancing the quality of the epidemiological data recorded. In light of modern concepts of caries management, the utility of the national registries and national surveys in Norway and Sweden is limited. For appropriate oral health planning in an organised dental service, reporting of enamel caries is essential.

## Competing interests

The authors declare that they have no competing interests.

## Authors’ contributions

Both authors contributed to the study idea and to the paper in general. MSS: contributed substantially to the manuscript writing. KSK: actively involved in the development of the study idea and provided valuable comments. Both authors have read and approved the final manuscript.

## Pre-publication history

The pre-publication history for this paper can be accessed here:

http://www.biomedcentral.com/1472-6831/14/43/prepub
